# Landscape Genomics Reveals Divergent Adaptation Modes and Predicts Climate Vulnerability in Xinjiang Indigenous Sheep

**DOI:** 10.3390/ani16172673

**Published:** 2026-08-25

**Authors:** Peng Yang, Mengsi Xu

**Affiliations:** 1 Institute of Animal Husbandry and Veterinary Medicine, Xinjiang Academy of Agricultural and Reclamation Science, Shihezi 832000, China; 13031327620@163.com; 2 College of Animal Science and Technology, Shihezi University, Shihezi 832000, China

**Keywords:** indigenous sheep, landscape genomics, climate adaptation, adaptive loci, genetic offset, future climate scenario

## Abstract

Climate change increasingly endangers precious indigenous sheep germplasm resources distributed across diverse Chinese landscapes, and systematically decoding their polygenic climate-adaptive genetic mechanisms is essential for targeted breed conservation and long-term sustainable pastoral production. Whole-genome resequencing data from 93 individuals covering six representative local sheep breeds were analyzed in this work. After filtering highly collinear climate variables, three mature landscape genomic approaches were jointly applied to identify environment-linked gene variants, while two predictive metrics across ten CMIP6 future climate scenarios quantified each breed’s long-term adaptive risks. Six temperature- and water-related environmental factors jointly drove sheep population genetic differentiation, with temperature fluctuation indices showing markedly stronger explanatory power. Detected adaptive genes were significantly enriched in ion transport, energy metabolism and cellular stress response pathways. Future projections indicated western breeds (Bayinbuluke, Cele Black, Xiahe) face severe maladaptation risks under high-emission SSP370 scenarios by 2100, whereas central and eastern breeds possess much broader climate tolerance. This study systematically reveals the core genomic basis of ovine climate adaptation and quantifies distinct breed-specific climate vulnerability, providing solid reliable theoretical support for precision germplasm conservation and selective breeding of climate-resilient sheep varieties.

## Simple Summary

Global warming threatens native sheep breeds that have adapted to unique local climates, yet we still lack clear knowledge about how these sheep adjust at the genetic level and how well they will cope with hotter, drier weather in the future. This study collected genetic material from six types of local sheep living in plateau, desert and grassland areas to find gene patterns linked to temperature and moisture conditions, and forecast their ability to survive future climate shifts. We found that changes in sheep genes are mainly shaped by year-round temperature swings, followed by seasonal rainfall and dryness. Multiple key genes that help sheep handle drought, cold and low oxygen were identified. Predictions show that sheep from dry western regions will struggle much more with future extreme weather, while breeds from central and eastern areas can tolerate wider climate changes. Our findings offer practical guidance to protect vulnerable local sheep varieties and breed climate-resilient sheep, supporting stable sheep farming as the planet’s climate keeps changing.

## 1. Introduction

Over the past several decades, a broad spectrum of extreme environmental stressors, which encompass hypoxic conditions, hyperthermic exposure, chronic cold stress and persistent aridity, impose robust selective forces that iteratively reshape the population-wide genetic architecture of domesticated livestock, a process that consequently generates substantial existential risks to the sustained conservation of indigenous livestock germplasm reservoirs [1]. Livestock lineages have accrued remarkably abundant phenotypic polymorphism and genetic variation over millennia of domestication, structured artificial selective breeding and ongoing natural environmental filtering, a composite evolutionary process that confers these taxa with marked adaptive plasticity enabling sustained reproductive viability amid heterogeneous harsh ecological niches [2]. Against the backdrop of steadily worsening climate disturbances, including global warming and erratic rainfall patterns, dissecting livestock’s molecular genetic regulatory mechanisms of climatic adaptation delivers both theoretical insights and practical strategies to enhance livestock stress resilience and support sustainable animal husbandry [3].

As one of the earliest domesticated ungulates, sheep (*Ovis aries*) constitute a paradigmatic livestock taxon that possesses prominent tolerance to multifarious climatic stressors, which manifests as conspicuous inter-population divergences across morphological, physiological and behavioral phenotypic profiles [4]. China encompasses vast stretches of ecologically fragile landscapes, which cover northern arid and semi-arid belts, hilly southwestern territories, the Qinghai–Tibet Plateau, and aquatic–terrestrial transitional zones distributed along eastern coastal corridors [5]. Subjected to prolonged natural selective pressures across such disparate ecological milieus, indigenous Chinese sheep have evolved specialized adaptive competencies against complex local climatic constraints, which establishes them as an optimal model system for decoding genetic variation and molecular regulatory cascades that govern organismal acclimatization to variable climates [6]. Geographically segregated sheep cohorts have evolved divergent adaptive characteristics, ranging from hypoxic resistance to cold and drought tolerance: plateau populations attain hypoxia resilience through synergistic physiological remodeling and heritable genetic alterations [7]; desert strains develop species-specific reproductive rhythms synchronized to periodic precipitation oscillations; cold-adapted breeds depend on rewired metabolic networks and modified somatic architectures to sustain viability under subzero thermal regimes [8]. Apart from overt phenotypic disparities captured by field monitoring, genome-wide scanning efforts have pinpointed a panel of candidate genes functionally coupled with climatic acclimatization [6]. To elaborate, *PCLB1* and *PCLB2* alongside *CAMK2D* orchestrate glucose metabolic homeostasis under arid circumstances; *EPAS1*, *IGF2BP2* and *MITF* jointly mediate high-altitude hypoxia tolerance, angiogenic processes and systemic energy balance; *SOX6*, *BMP2* and *TSHR*, by contrast, participate in core metabolic cascades and execute central regulatory functions governing cellular responses to warm ambient temperatures. However, approaches relying on phenotypic monitoring and candidate genotyping fail to disentangle environmental and genetic effects, especially for polygenic traits controlled by multiple loci [9].

With the rapid advancement of landscape genomics, genome–environment association analytical approaches including gradient forest, redundancy analysis (RDA) and latent factor mixed model (LFMM) have evolved into core methodologies for excavating genetic variants underlying environmental adaptation in animals, which enable the precise identification of functional loci and gene pathways under selective pressures exerted by climatic factors [10]. On this basis, the present study recruited a total of 93 individuals covering six indigenous sheep breeds distributed across distinct ecological zones in China as research subjects, leveraged whole-genome resequencing datasets and integrated multiple association algorithms to screen single-nucleotide polymorphisms (SNPs) and functional genes associated with climatic acclimatization. Furthermore, this study incorporated ten CMIP6 future climate scenarios and adopted risk of non-adaptedness (RONA) as well as genetic offset metrics to quantify the maladaptation risks across various sheep populations. The current work aims to decipher the molecular genetic basis governing climatic adaptation in Chinese indigenous sheep, disentangle adaptive discrepancies among sheep ecotypes, and deliver robust theoretical underpinnings for the conservation, administration and selective breeding of ovine genetic resources amid progressive climate change.

## 2. Materials and Methods

### 2.1. Ethical Approval

The experimental design and animal management protocol were reviewed and approved by the Animal Care and Use Committee of the Xinjiang Academy of Agricultural and Reclamation Sciences (Xinjiang, China, approval number: XAARS-IACUC-2026-021, approval date: 15 February 2026), and all procedures complied with its guidelines for the care and use of experimental animals.

### 2.2. Sample Information and Genomic Data Statistics

The whole-genome resequencing data utilized in the present study were derived from a previously completed research project of our team, whose raw sequencing datasets have been deposited in the National Genomics Data Center, China National Center for Bioinformation (CNCB-NGDC, https://ngdc.cncb.ac.cn) under the project accession number PRJCA052911. A total of six indigenous sheep breeds that represent typical germplasm resources from distinct ecological regions in China were selected as the research materials in this study, and all sampled individuals were confirmed to be genetically unrelated through the comprehensive verification of local breeding records and population pedigree information (Table S1). Specifically, a total of 45 Xiahe sheep (abbreviated as XH) were collected from the Xiahe Forest Farm of the Third Division, while 10 Bayinbuluke sheep (BYBLK) were sampled from the pastoral areas covering the Bayinbuluke Grassland and the Kaidu River Basin. In addition, 10 Cele Black sheep (CLH) were obtained from the northern foot of the Kunlun Mountains and the southern margin of the Taklamakan Desert, and Sunite sheep (SNT) were collected from the Inner Mongolia Plateau located to the northwest of Daqing Mountain. To expand the sample size and maximize the utilization efficiency of existing genomic data, publicly available whole-genome sequencing datasets of sheep were further retrieved from the National Center for Biotechnology Information database (www.ncbi.nlm.nih.gov), which were subjected to strict quality control and systematic screening based on the sample-size suitability evaluation to supplement the genomic data of Hu sheep (HU), SNT and Small-tailed Han sheep (STH) (Table S2). Figure S1 presents high-quality photographs of each studied breed (Figure S1a) and a geographical map of the study area (Figure S1b).

Ultimately, a comprehensive dataset containing whole-genome sequencing information from 93 individuals across six sheep breeds was compiled in this study, including 45 XH, 10 BYBLK, 10 CLH, 10 HU, 9 SNT, and 9 STH [11]. Since all experimental samples originate from diverse typical ecological environments involving plateaus, grasslands, deserts and plains, the compiled dataset is sufficiently qualified for subsequent analyses focused on the genetic characteristics and adaptive evolutionary mechanisms of different sheep germplasms. To alleviate analytical bias caused by unequal sampling depth, we implemented strict pre-processing standards for all populations uniformly: all individuals were confirmed genetically unrelated via complete breeding records and pedigree validation before inclusion; identical multi-step SNP filtering thresholds were applied to every breed’s genomic dataset without population-specific parameter adjustment; allele frequencies were calculated on per-breed independent cohorts rather than pooled whole-population matrices to avoid over-weighting the XH group in environmental association statistics.

### 2.3. SNP Identification, Filtering and Annotation

In this study, fastp (v 0.23.4) software with the parameters of --trim_front 15 --trim_tail 15 --trim_front 25 --trim_tail 25 --cut_right --cut_window_size 4 --cut_mean_quality 20 --length_required 50 --n_base_limit 3 --qualified_quality_phred 20 --unqualified_percent_limit 10 was utilized to perform rigorous quality control and sequence trimming on raw sequencing data as well as publicly available sheep sequencing datasets (Table S3), and the yielded clean reads were aligned to the Ovis aries Oar_v4.0 reference genome utilizing bwa-mem2 v2.2.1 via index and mem commands to generate primary BAM files. The obtained BAM files were sorted and indexed by Samtools v1.19.2 for standardized preprocessing. Subsequently, bcftools v1.19 was used to identify SNP and InDel variants for each sample, and all individual BCF files were merged to construct the initial population genomic variation dataset.

To ensure the accuracy and reliability of subsequent population genetic analyses, multi-step stringent filtering strategies were performed on the raw SNP dataset derived from 93 sheep individuals. First, an initial filtering procedure was performed on the raw variant calls using bcftools, which excluded SNPs that exhibited a missing rate exceeding 30%, a zero minor allele count, or a minor allele frequency below 0.05, as well as those that were located within 3 bp upstream and downstream of InDel loci. This preliminary filtering step was performed to remove highly incomplete variants from the raw dataset and reduce computational burden before subsequent dataset integration and more stringent quality control. For dense InDel clusters where the inter-locus interval was less than 10 bp, only the InDel variant with the highest quality score within each cluster was preserved. After format conversion and integration of the filtered SNP dataset, Plink 1.9 was adopted for secondary quality control. Although the same MAF threshold was applied, Plink recalculated allele frequencies based on the complete integrated dataset of 93 sheep individuals and performed additional filtering procedures. A stricter genotype missingness threshold (geno < 0.05) was subsequently applied to obtain a high-confidence SNP dataset for downstream analyses. Plink screening reserved exclusively biallelic SNPs while removing variants that failed to satisfy the thresholds of MAF < 0.05 and genotype missing rate < 0.05. Variants that showed significant deviations from the Hardy–Weinberg equilibrium at *p* < 0.001 were also discarded. Moreover, linkage disequilibrium pruning that adopted a 10 kb window, a single-SNP step size, and an r^2^ threshold of 0.2 was implemented to eliminate redundant SNPs with strong linkage disequilibrium. After systematic quality control and hierarchical filtering, the final high-quality SNP dataset was acquired to support subsequent population genetic analyses.

### 2.4. Collection of Bioclimatic Variable Data

Historical bioclimatic data that covers the time period from 1970 to 2000 were retrieved from the WorldClim 2.1 database (https://worldclim.org/), which provides 19 standard bioclimatic variables (BIO1–BIO19) with a spatial resolution of 30 arcseconds. Meanwhile, 11 topoclimatic variables that reflect current environmental conditions were obtained from the ENVIREM database, which encompasses a series of key metrics including annual potential evapotranspiration (annualPET), Thornthwaite aridity index (aridityIndexThornthwaite), climatic moisture index (climaticMoistureIndex), and Continentality index (continentality) [12].

To eliminate multicollinearity among environmental predictors, we calculated the Pearson correlation coefficients for all 30 environmental variables, among which only the variables with absolute correlation coefficients no more than 0.7 were retained for subsequent statistical analysis (https://popgen.nescent.org/2018-03-27_RDA_GEA.html, accessed on 4 August 2025). Ultimately, six core environmental factors that dominate the local environmental variation were screened out for further investigation, which include Thornthwaite aridity index (aridityIndexThornthwaite), Precipitation of the driest month (BIO14), Precipitation seasonality (BIO15), Continentality, minimum temperature of the warmest month (minTempWarmest), and potential evapotranspiration of driest quarter (PETDriestQuarter) (Table 1).

Future bioclimatic datasets utilized for scenario prediction were obtained from the WorldClim CMIP6 database (https://worldclim.org/data/cmip6_all/cmip6climate.html, accessed on 16 August 2025), from which three representative global climate models (ACCESS-CM2, BCC-CSM2-MR, and GISS-E2-1-G) and two identical shared socioeconomic pathways (SSP126 and SSP370) across two future temporal horizons (2061–2080 and 2081–2100) were selected for systematic data acquisition. Complementary future ENVIREM topoclimatic variables, which function as essential auxiliary predictors for ecological modeling, were derived from the matched future bioclimatic datasets via the standardized ENVIREM computational pipeline.

### 2.5. Gradient Forest (GF) Analysis

Gradient Forests (GF) analysis was implemented with the gradientForest package (v 0.1) [13] to assess nonlinear impacts of environmental factors on population genetic variation. This method combines univariate random forest analysis and cross-validation of importance scores to evaluate the explanatory power of environmental variables and quantify the fraction of genetic variation they account for. Allele frequencies of six sheep breeds and six core environmental predictors served as analytical inputs. Factor importance was weighted by the coefficient of determination(R^2^). We calculated the contribution proportion of each predictor along environmental gradient components to derive the absolute and relative importance values of environmental factors; larger values denoted stronger ability to explain the genetic variation in response variables.

### 2.6. RDA-Based Candidate Loci Screening and Pathway Enrichment Analysis

To clarify how environmental variables independently modulate biological adaptability, a partial RDA was carried out. The allelic frequency matrix derived from each sample was treated as the response variable, and six environmental indicators were incorporated as explanatory variables for this model. Partial RDA analyses were implemented utilizing the vegan (v 2.5.2) package [14], followed by permutation tests to quantify the statistical significance of these environmental variables. The variance inflation factor (VIF) for each environmental variable explained by separate RDA axes was computed to evaluate multicollinearity across environmental factors. To screen candidate adaptive SNPs and reduce false positives from ordination results, we retained variants with loading values exceeding three standard deviations on constrained RDA axes as putative adaptive loci. For each retrieved SNP, pairwise Pearson correlation coefficients against all environmental variables were estimated, and the environmental factor yielding the highest absolute correlation coefficient was identified as its primary correlative factor. After extracting candidate SNPs residing within gene regions, Gene Ontology (GO) enrichment analysis was performed on their adjacent genes, wherein Fisher’s exact test was utilized to determine the significance of individual GO terms at a conventional cutoff of *p* < 0.05.

### 2.7. LFMM-Based Candidate Loci Screening and Pathway Enrichment Analysis

To unravel the genomic regions associated with environmental adaptation in the admixed XH population, the univariate LFMM from the LEA (v 3.14.0) package [15] was adopted in the present study. Given that four ancestral populations (K = 4) were inferred utilizing ADMIXTURE, we set four latent factors to mitigate the impacts of population structure on genotype datasets. Cross-validation error across K = 1–10 confirmed K = 4 as the optimal number of neutral genetic clusters, which allows LFMM latent terms to fully absorb genome-wide neutral population stratification and effectively filter out false-positive SNP–environment associations caused by demographic divergence (Figure S2a). Each environmental variable was analyzed through five independent Markov chain Monte Carlo (MCMC simulations), with 5000 iterations assigned as the burn-in phase and an additional 10,000 iterations for subsequent sampling. The mean *p*-values calculated from all five runs were regarded as the ultimate association *p*-values, and variants with a false discovery rate (FDR) < 0.05 were retained as candidate loci. *p*-values from five independent LFMM runs were averaged, and Benjamini–Hochberg FDR correction was performed with q < 0.05 defined as the significance threshold for identifying candidate loci. After overlapping the LFMM-derived SNPs with candidate SNPs from RDA, we acquired a set of robust candidate loci and conducted GO enrichment analysis on these variants (*p* < 0.05).

### 2.8. RONA Under Future Climate Scenarios

RONA is a metric used to quantify the mean allele frequency alteration of environmentally associated SNPs across future climate scenarios, serving as an indicator of potential maladaptation risks [16]. Based on the environment-adaptive SNPs and their allele frequencies obtained from LFMM analysis, RONA was computed for six sheep breeds utilizing the pyRona (v 0.4.4) package [17]. Populations with higher RONA values need more substantial genetic modifications to cope with future climate changes and thus possess weaker adaptive capacity.

We adopted 10 future climate scenarios for prediction, which were generated from three climate models, two SSPs and two time horizons. These scenarios included ACCESS-CM2_SSP370 (2061–2080, 2081–2100), BCC-CSM2-MR_SSP126 (2061–2080, 2081–2100), BCC-CSM2-MR_SSP370 (2061–2080, 2081–2100), GISS-E2-1-G_SSP126 (2061–2080, 2081–2100) and GISS-E2-1-G_SSP370 (2061–2080, 2081–2100).

### 2.9. Generalized Dissimilarity Modelling (GDM)

GDM is a powerful approach that fits biological distances against pairwise geographic and environmental distances, and has been widely applied to quantify genetic shifts along geographic and environmental gradients [18]. Referencing the analytical framework established by Jia et al. (2022) [19], GDMs were separately built for each climate model via the gdm (v 1.6.0-7) package [20]. With pairwise Fst and geographic coordinates as analytical datasets, genetic offset values of sheep breeds were calculated to reflect genetic changes under various future climate scenarios [21].

## 3. Results

### 3.1. Environmental Factors Driving Genetic Differentiation in Sheep

**Figure 1:**
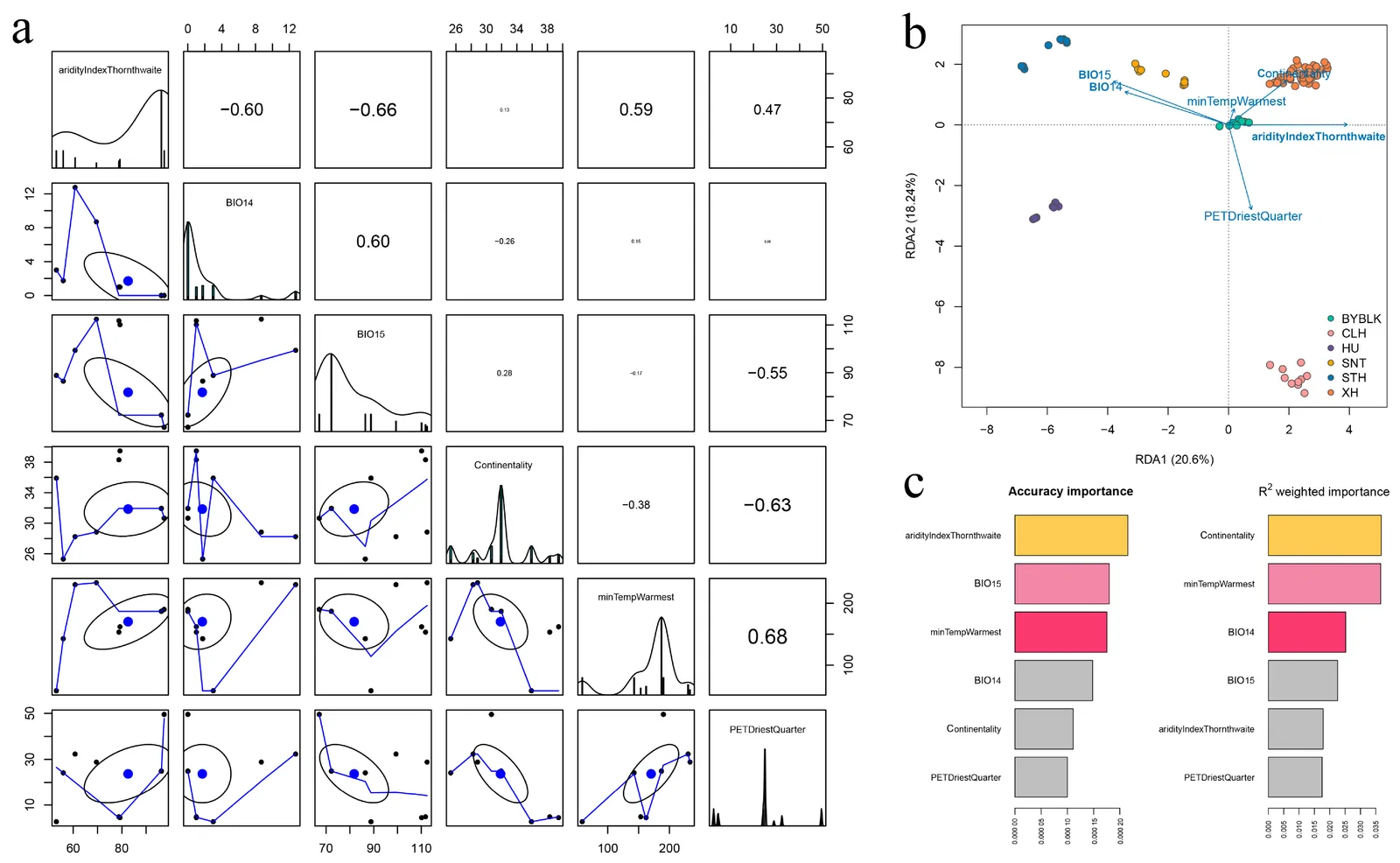
Environmental factors and genetic variation in sheep breeds. (**a**) Correlation matrix of environmental variables after collinearity pruning (|r| ≤ 0.7). (**b**) RDA ordination showing the relationship between six environmental variables and genetic variation. (**c**) Variable importance from gradient forest analysis (R^2^-weighted).

A correlation matrix of six pre-filtered environmental factors was generated to reduce multicollinearity and improve the stability of downstream analyses (Figure 1a). The retained variables covered two ecological dimensions: water availability, including aridityIndexThornthwaite, BIO14, BIO15 and PETDriestQuarter, and temperature variability, represented by Continentality and minTempWarmest. A strong positive correlation was detected between BIO14 and BIO15 (r = 0.60), while aridityIndexThornthwaite was negatively correlated with BIO14 (r = −0.60) and BIO15 (r = −0.66).

With the low-collinearity environmental variables obtained, we further performed partial RDA to explore the associations between environmental gradients and population genetic structure. The first two axes explained 38.84% of total genetic variation (20.60% for RDA1 and 18.24% for RDA2; Figure 1b). Continentality and minTempWarmest were the main contributors to RDA1, and BIO14 and BIO15 dominated RDA2. All six constrained axes collectively explained 100% of the constrained genetic variation and were statistically significant (*p* < 0.001, 999 permutations, Table 2), confirming that the selected environmental factors collectively exert strong explanatory power on the population genetic composition of the studied sheep breeds.

All six sheep breeds formed discrete clusters in the ordination plot, consistent with environment-associated genetic differentiation, although such clustering may also arise from other factors including population demographic history, geographic isolation, artificial selection and breeding management practices (Figure 1b). XH was positioned along the positive axes of both aridityIndexThornthwaite and Continentality. SNT and STH were strongly associated with precipitation seasonality variables (BIO14 and BIO15), while CLH showed a high correlation with PETDriestQuarter. BYBLK exhibited associations with aridityIndexThornthwaite, Continentality, and PETDriestQuarter. Notably, HU was separated from the main environmental gradients, potentially reflecting adaptation to intermediate or the influence of distinct population histories and breeding backgrounds.

To further quantify the relative explanatory capacity of each environmental factor for observed genetic variation, GF analysis was conducted (Figure 1c). Continentality ranked first, followed by minTempWarmest, BIO14, BIO15, aridityIndexThornthwaite and PETDriestQuarter. These results demonstrate that both temperature and water availability related factors collectively drive adaptive genetic differentiation across the studied sheep populations.

Finally, significance tests revealed that all six environmental variables significantly contributed to genetic variation (df = 1, 999 permutations, *p* < 0.001), indicating strong independent explanatory effects of each factor (Table 3). While aridityIndexThornthwaite (VIF = 165.07) and minTempWarmest (VIF = 88.72) exhibited relatively high VIF, suggesting partial collinearity with other variables, PETDriestQuarter showed the lowest VIF value (4.21), reflecting minimal collinearity and the highest degree of independence among the tested factors. Residual multicollinearity could bring subtle uncertainty to individual predictor effect estimates. Accordingly, our biological interpretations centre on overall ordination patterns and relative variable importance rather than precise numerical differences between predictors.

### 3.2. Landscape Genome and Environmental Association Analysis

To elucidate the core genetic loci that are tightly correlated with environmental variables, genotype–environment association (GEA) approaches including RDA and LFMM were adopted to screen and characterize SNPs underlying climate adaptation in the studied species. The RDA-based analytical results demonstrated that the PETDriestQuarter was associated with the largest number of candidate SNPs (3637), followed by Continentality (1653 SNPs), BIO14 (1008 SNPs), aridityIndexThornthwaite (842 SNPs), BIO15 (668 SNPs), and minTempWarmest (314 SNPs), as summarized in Table 4.

We then performed a genome-wide association analysis between six core environmental factors and whole-genome SNPs utilizing the LFMM. SNPs significantly associated with environmental variables were screened based on q-values derived from FDR correction. As presented in Table 5, a total of 1204 SNPs were associated with the aridityIndexThornthwaite, 1368 SNPs with BIO14, 912 SNPs with BIO15, 2156 SNPs with PETDriestQuarter, 1107 SNPs with Continentality, and 708 SNPs with minTempWarmest. Subsequent genomic localization of these candidate SNPs enabled the successful annotation of a total of 1423 functional genes.

**Figure 2:**
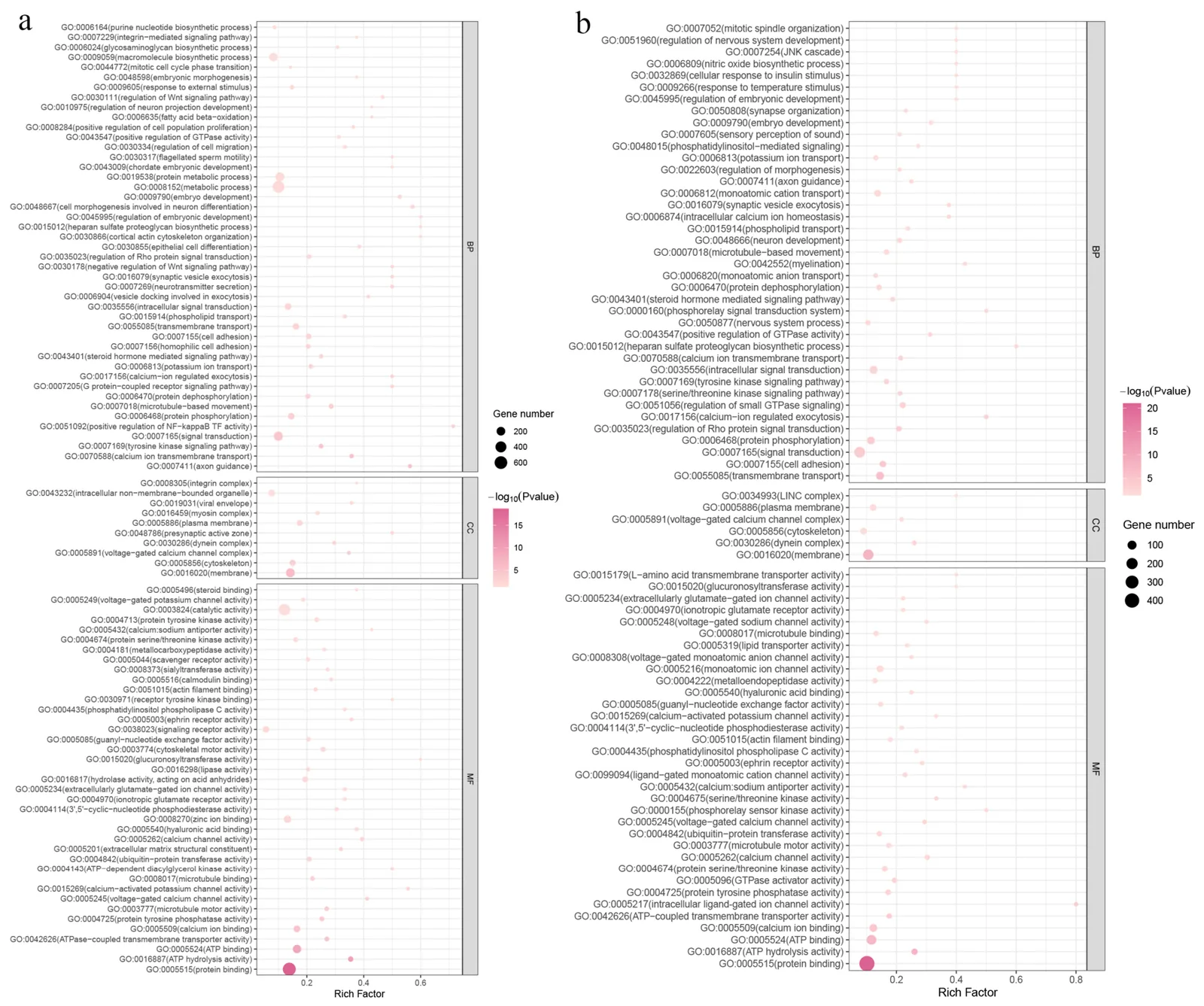
GO enrichment of environment-associated SNPs. (**a**) GO enrichment for genes linked to RDA candidate SNPs. (**b**) GO enrichment for genes linked to LFMM candidate SNPs. Dot size indicates the number of enriched target genes within each GO term, and the gradient color of dots corresponds to −log_10_(*p* value), where higher values denote more significant enrichment.

Based on the aforementioned datasets, gene enrichment analysis was further conducted for the candidate genes linked to the SNPs identified by RDA and LFMM, respectively (Figure 2a). In total, 8122 candidate SNPs were captured by the RDA pipeline, while 5713 candidate SNPs were detected via LFMM analysis. We identified 725 overlapping SNPs that were associated with identical environmental predictors in both analytical frameworks. QQ plots for LFMM analyses (Figure S2b) yielded genomic inflation factors λ between 0.920 and 0.965 for the six environmental variables. Observed test statistics deviated above the null expectation, consistent with the presence of environment-associated SNPs. λ values near unity suggest limited inflation induced by population stratification, supporting reliable association results. Notably, the functional genes associated with SNPs from both two analytical methods were predominantly enriched in multiple identical GO terms, which encompassed protein binding (GO: 0005515, 466 genes), ATP binding (GO: 0005524, 126 genes), calcium ion binding (GO: 0005509, 64 genes), ATPase-coupled transmembrane transporter activity (GO: 0042626, 13 genes), and protein tyrosine phosphatase activity (GO: 0004725, 15 genes). In addition, the LFMM approach uniquely identified two additional significantly enriched GO terms that were not detected in the RDA (Figure 2b), namely calcium channel activity (GO: 0005262, 10 genes) and voltage-gated calcium channel activity (GO: 0005245, 5 genes), which indicates that the LFMM method possesses complementary advantages in the identification and detection of genes related to ion channel functions. Furthermore, a total of 2847 overlapping candidate SNPs corresponding to 673 associated genes were jointly identified by both RDA and LFMM methods.

### 3.3. Genomic Loci Associated with Climatic Variables

**Figure 3:**
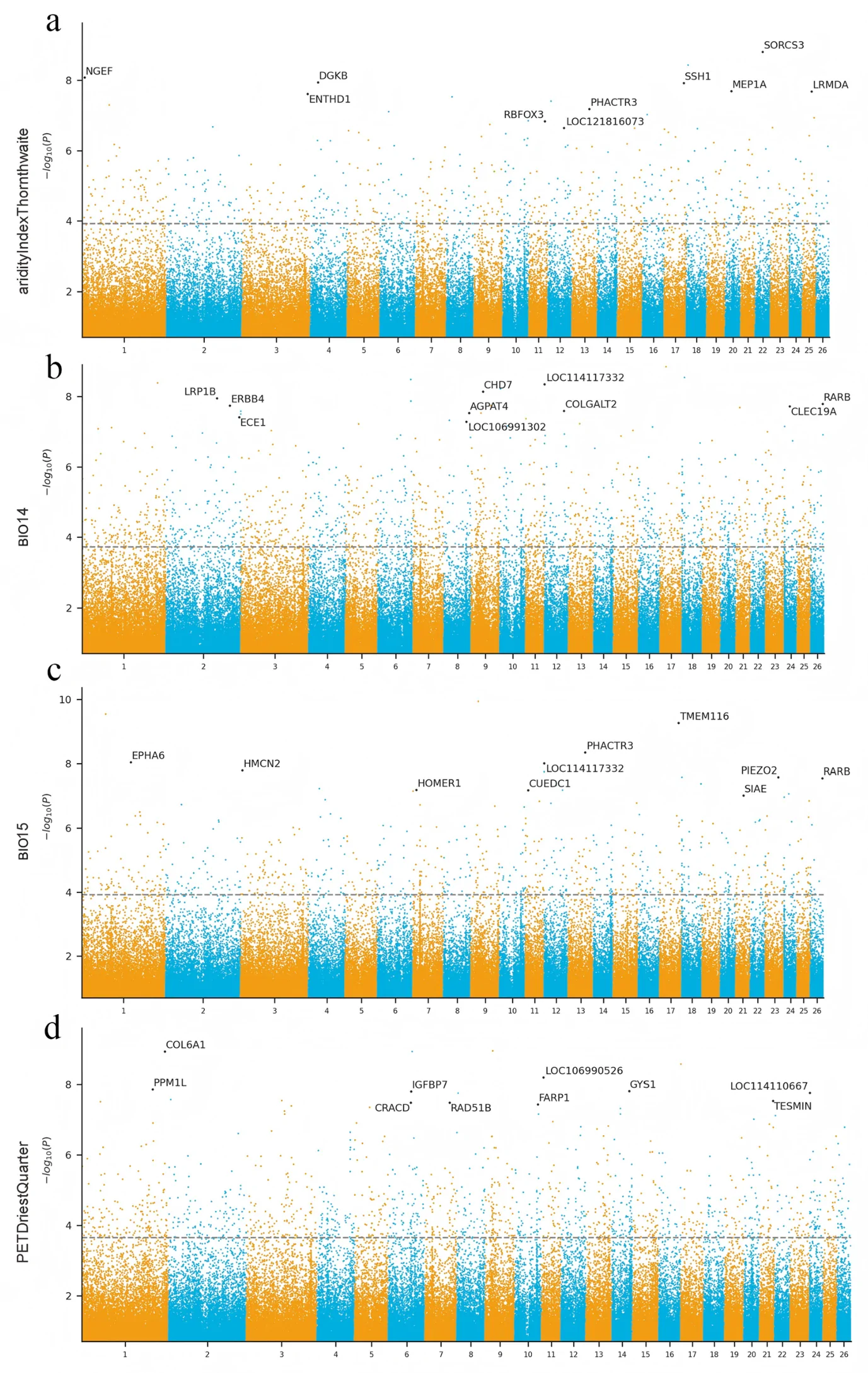
LFMM Manhattan plots for water-related environmental factors. Manhattan plots showing −log_10_(*p* values) for SNPs associated with (**a**) aridityIndexThornthwaite, (**b**) BIO14, (**c**) BIO15, and (**d**) PETDriestQuarter. Yellow dots represent all SNPs from LFMM analysis, and blue dots indicate candidate SNPs jointly identified by both LFMM and RDA analyses. The dashed line marks the FDR < 0.05 significance threshold.

We further identify loci associated with four water availability-related environmental variables in XH: the aridityIndexThornthwaite, BIO14, BIO15, and PETDriestQuarter. Manhattan plots revealed multiple genomic loci significantly associated with these variables at the FDR-corrected threshold (*p* < 0.05; −log_10_(*p*) ≈ 4). Under the Thornthwaite aridity index, the strongest associations were observed at loci containing *SORCS3* and *URMDA*, both exceeding −log_10_(*p*) ≈ 8, alongside other notable genes including *NGEF*, *DGKB*, *ENTHO1*, *RBFOX3*, *PHACTR3*, *SSH1*, and *MEP1A* (Figure 3a). For BIO14, significant associations were detected for genes such as *LRP1B*, *ERBB4*, *ECE1*, *AGPAT4*, *COL4A3/2*, *CLEC19A*, and *RARB*, with the latter two showing particularly high significance (−log_10_(*p*) ≈ 8, (Figure 3b). BIO15 exhibited the strongest association signal at *TMEM116* (−log_10_(*p*) ≈ 10), with additional significant loci including *EPHA6*, *HCN2*, *HOMER1*, *PIEZO2*, *SUAE*, and *RARB* (Figure 3c). Under PETDriestQuarter, prominent associations were found at *COL6A1*, *KGFBP7*, and *PPM1L* (−log_10_(*p*) ≈ 8), as well as *CRACO*, *NAD518*, *FARP1*, *GYS1*, and *TESMIN* (Figure 3d).

**Figure 4:**
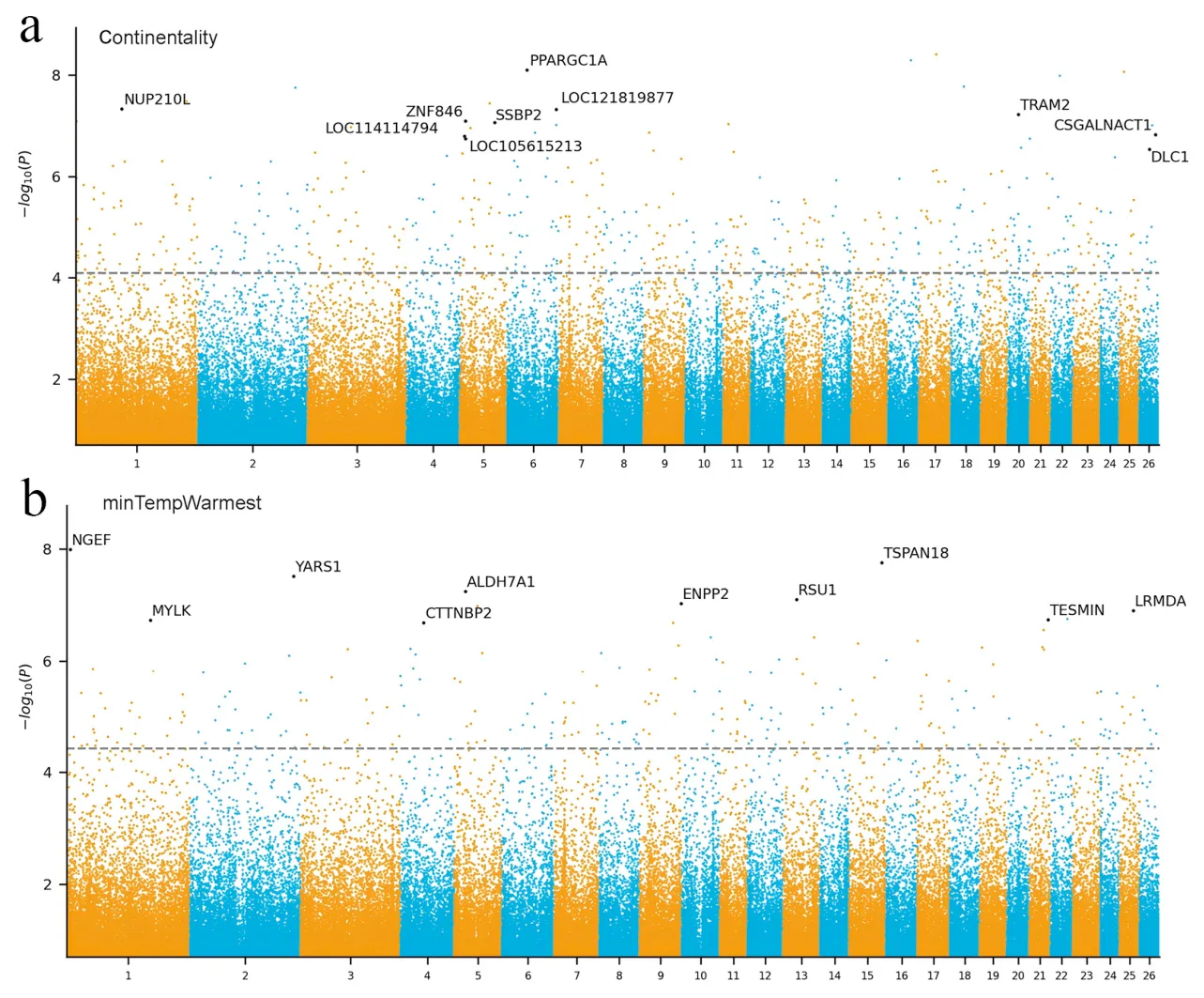
LFMM Manhattan plots for temperature-related environmental factors. Manhattan plots showing −log_10_(*p* values) for SNPs associated with (**a**) Continentality and (**b**) minTempWarmest. Yellow dots represent all SNPs from LFMM analysis, and blue dots indicate candidate SNPs jointly identified by both LFMM and RDA analyses. The dashed line marks the FDR < 0.05 significance threshold.

For temperature variability factors (Continentality and minTempWarmest), significant associations were also detected in XH. Under Continentality, *PPARGC1A* showed the strongest signal (−log_10_(*p*) > 8), alongside genes such as *NUP210L*, *TRAM2*, and *DLC1* (Figure 4a). For minTempWarmest, prominent associations were found at *NGEF*, *YARS1*, *ALDH7A1*, *TSPAN18*, and *LRMDA*, with some genes (e.g., *NGEF*) also linked to water availability, suggesting multifaceted roles in climatic adaptation (Figure 4b). Collectively, these results reveal polygenic adaptation to climate, with distinct but overlapping genomic regions associated with water and temperature-related selection pressures.

### 3.4. Spatiotemporal Patterns of Projected Climatic Variables

We characterized the spatiotemporal patterns of six key climatic variables—aridityIndexThornthwaite, BIO14, BIO15, Continentality, minTempWarmest, and PETDriestQuarter—across the study region under 10 future climate scenarios and six sheep breeds (Figures S3–S5). Under the high-emission SSP370 scenario, the aridityIndexThornthwaite and PETDriestQuarter exhibited pronounced increases across most models, particularly in the western and central habitats of BYBLK and CLH, with the most severe aridification projected in ACCESS-CM2 simulations for 2081–2100 (Figure S3b), where aridityIndexThornthwaite rose by up to 0.16 relative to the baseline. Concurrently, BIO15 showed a consistent upward trend across scenarios, reflecting amplified intra-annual precipitation variability, which was most evident in the eastern range of SNT in GISS-E2-1-G projections for the 2081–2100 period (Figure S5d), with values exceeding 175. Temperature-related variables, including Continentality and minTempWarmest, displayed spatially heterogeneous changes; Continentality increased notably in the northern and western zones inhabited by XH and BYBLK, whereas the minimum temperature of the warmest month rose most sharply in the southeastern habitats of HU and STH, especially under BCC-CSM2-MR and GISS-E2-1-G SSP370 simulations (Figures S4d and S5d). BIO14 remained relatively stable or declined slightly across most scenarios, with only minor increases observed in a few eastern sites of SNT under SSP126 (Figures S4a and S5a). Notably, model agreement was higher for temperature-related variables than for precipitation-derived indices, with SSP370 consistently driving more extreme climatic shifts than SSP126, and the later period (2081–2100) exhibiting amplified trends relative to the earlier 2061–2080 horizon. Collectively, these projections indicate that all investigated breeds will face escalating aridity, heightened precipitation seasonality, and greater temperature variability under future climate change, with the most severe impacts expected under high-emission scenarios by the end of the century.

### 3.5. Genetic Offset Analysis of Adaptive Vulnerability Under Future Climate Scenarios

**Figure 5:**
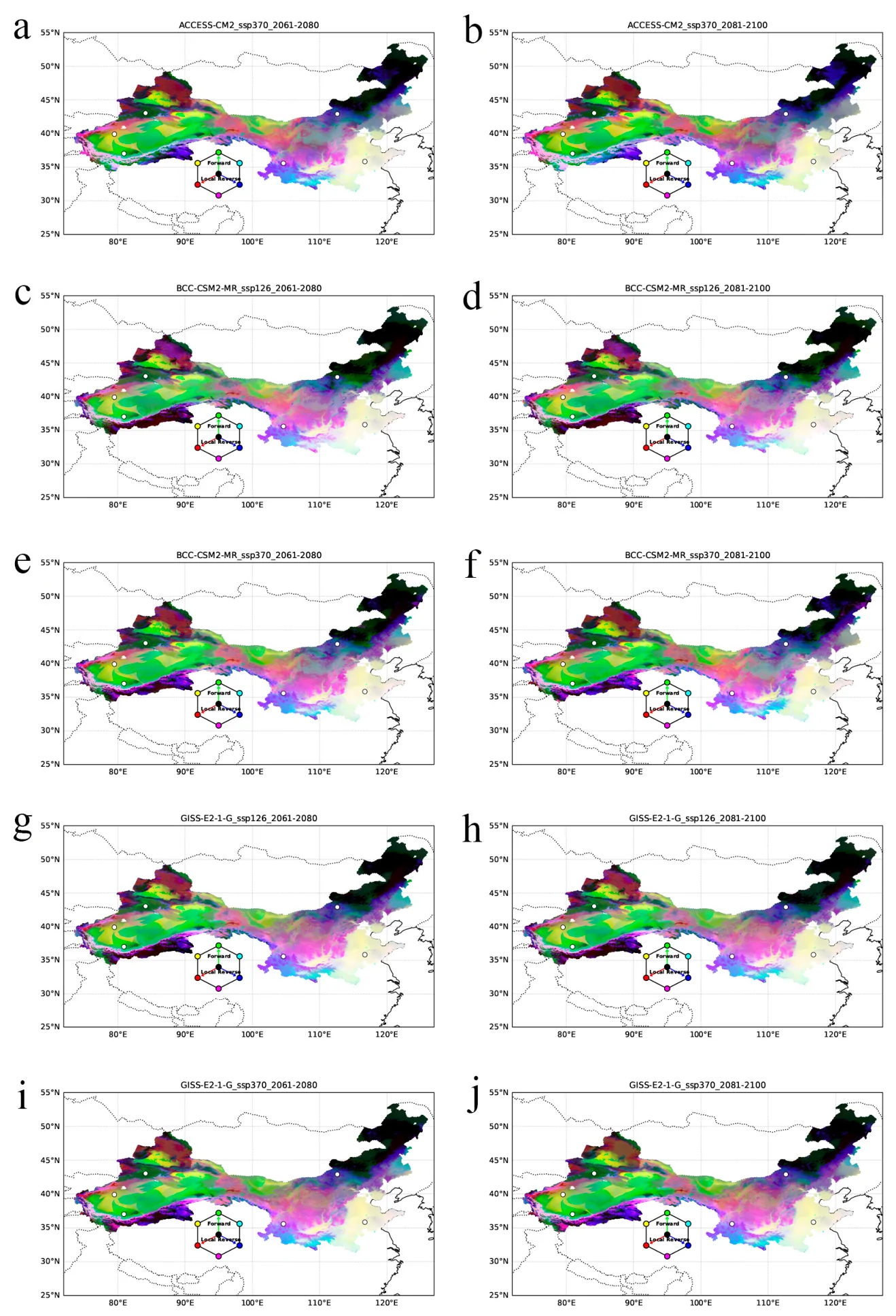
RONA analysis under 10 future climate scenarios. RONA values reflect risk of non-adaptedness. Subplots: (**a**,**b**) ACCESS-CM2 SSP370; (**c**,**d**) BCC-CSM2-MR SSP126; (**e**,**f**) BCC-CSM2-MR SSP370; (**g**,**h**) GISS-E2-1-G SSP126; (**i**,**j**) GISS-E2-1-G SSP370. Time periods: 2061–2080 (**a**,**c**,**e**,**g**,**i**) and 2081–2100 (**b**,**d**,**f**,**h**,**j**). Inset hexagonal plots summarize forward and backward climatic transfer risks across sampling sites within each panel. RGB colours denote local (red), forward (green), and backward (blue) genetic offset; brighter (near-white) cells indicate high values on all three axes, and darker (near-black) cells indicate low values. Colour gradients in the main map represent continuous RONA values, with higher colour intensity indicating greater non-adaptation risk.

We further evaluated the adaptive vulnerability of the studied sheep breeds by performing genetic offset analyses under the aforementioned future climate scenarios (Figure 5). As illustrated by the RGB composite maps, substantial spatial heterogeneity existed in the risk of genetic maladaptation, where blended coloration denoted the superposition of multiple offset signals. Under the 2061–2080 near-term projection period (Figure 5a,c,e,g,i), preliminary spatial differentiation of genetic offset was already detectable across the study area, in which western populations (BYBLK, CLH, XH) presented prominent red and purple coloration, which corresponded to elevated local and comprehensive genetic offsets, especially under the SSP370 scenario during 2081–2100 (Figure 5b,f,j). Such a finding suggests that these western breeds have formed narrow adaptive niches that are highly dependent on distinctive local microclimates, such as cool high-altitude habitats and extremely arid surroundings, which in turn restrict their genetic and phenotypic flexibility to cope with ongoing rapid climate changes. In comparison, populations distributed across eastern and central regions (SNT, HU, STH) showed relatively weak offset signals accompanied by dominant green and blue hues in partial areas (Figure 5c,g), a phenomenon that demonstrates their superior adaptive potential and broader climatic tolerance likely shaped by long-term evolution under fluctuating environmental conditions. It is also noteworthy that genetic offset values were universally higher under the high-emission SSP370 pathway than under SSP126, and that such adverse trends became more pronounced in the later period (2081–2100) relative to the earlier timeframe (2061–2080). Taken together, these results demonstrate that accelerating climate change will continuously aggravate adaptive mismatches between genomic makeup and ambient environments. In particular, the arid and semi-arid western habitats, where BYBLK, CLH and XH reside, will witness the most severe maladaptation risks by the end of this century.

Furthermore, RONA was calculated separately for each CMIP6 climate model, which quantifies projection uncertainty among GCMs (Figure S6). Population-level RONA differed across environmental variables, emission scenarios (SSP126 vs. SSP370) and future time periods (2061–2080, 2081–2100). Generally, higher RONA was observed under SSP370 than SSP126 for most populations, particularly towards the late century, revealing intensified adaptive pressure under high-emission pathways.

## 4. Discussion

Deciphering ovine adaptation to diverse extreme environments advances evolutionary research and sustainable livestock production, so we integrated whole-genome resequencing and high-resolution environmental covariates to dissect genomic adaptive bases across ecological gradients. Our combined pRDA and GF analyses indicate that sheep genetic differentiation is jointly driven by temperature variability and water availability, supporting polygenic adaptation under multi-climatic stress in ruminants living in complex habitats [22], as demonstrated by studies conducted by Liu et al. (2023) [22] and Jin et al. (2024) [6]. However, the first two pRDA axes merely explained 38.84% of genomic variance, meaning climate serves as a core but non-exclusive driver while geographic isolation and artificial selection also reshape population genetic structure [23]. Consistent with the GF outputs, temperature variability dominates ovine genetic divergence. This challenges the conventional view that arid species are primarily limited by water scarcity and highlights that drastic continental temperature fluctuations exert strong selective pressure on ovine thermoregulation and metabolism [24]. Moderate collinearity among temperature-related metrics points to synergistic thermal adaptation, which requires holistic ecological interpretation rather than oversimplified statistical inference. Meanwhile, water availability acts as a vital secondary driver for genomic differentiation. Precipitation seasonality shapes the secondary genetic gradient and covaries with the aridity index to structure genomic variation along aridity and precipitation-fluctuation [25]. Among water-related predictors, potential evapotranspiration of the driest quarter shows low collinearity and represents a robust water-balance indicator for arid-livestock genomic analyses, and the aridity index remains informative for identifying drought-adaptive signatures despite high VIF values [26]. This outcome aligns with earlier landscape-genomic work on ruminants reporting that continental temperature fluctuation, rather than aridity, acts as the primary selective filter for inland sheep populations [27]. Breed-level pRDA clustering reflects environment-associated local adaptation, although demographic history, geographic isolation, and artificial selection also contribute to population separation. Each breed displays genomic signatures corresponding to its native habitat: XH, originating from a warm-temperate continental arid zone with very low annual precipitation, shows strong genomic associations with aridity and continentality indices, matching its adaptation to severe drought and thermal fluctuation [28]. SNT and STH correlate with seasonal-precipitation variables, consistent with the precipitation regimes of the Tianshan steppe and semi-arid Inner Mongolia grassland. CLH and BYBLK harbour genomic signals compatible with arid high-evaporation environments [29], whereas HU from humid mid-lower Yangtze River regions is linked to dry-season evapotranspiration, reflecting humid-habitat adaptation [30]. These environment–genome correlations support a role for local adaptation in sheep breed divergence and phenotypic differentiation, offering theoretical support for germplasm conservation and climate-resilient breeding under global change [31,32].

To identify genome-wide SNPs underlying climatic adaptation driven by temperature variability and water availability, we integrated two complementary GEA strategies to reveal adaptive mechanisms enabling sheep survival under diverse ecological pressures. Our GEA results highlight the predominant contribution of dry-quarter potential evapotranspiration, followed by continentality and seasonal-precipitation metrics, to adaptive genomic variation. The dominant SNP signals associated with PETDriestQuarter indicate that seasonal water loss driven by evapotranspiration is a major hydrological selective pressure shaping ovine genomic variation [4], as prolonged drought promotes physiological adaptations including enhanced water retention, lipid storage, and epidermal protection against desiccation and ultraviolet radiation [33]. This pattern agrees with arid-land sheep adaptation, where seasonal evapotranspiration fluctuations impose stronger cumulative stress than static annual aridity, reshaping selective regimes across generations [34]. Continentality ranked second in associated SNP number, indicating that extreme temperature fluctuations impose evolutionary pressures comparable to drought. Alternations between hot summers and cold winters drive selection on genes involved in mitochondrial metabolism, thermogenesis, and cold tolerance [35], consistent with previous genomic studies identifying continental temperature variation as a major driver of sheep differentiation [27]. Protein-tyrosine-phosphatase-related pathways mediate cellular responses to combined aridity and temperature stress and modulate adipose thermogenesis and heat-stress tolerance in domestic ruminants, functioning as fine-tuning regulators for polygenic multi-stress adaptation [36,37]. Beyond shared adaptive signals, LFMM uniquely enriched calcium channel and voltage-gated calcium channel activity. Voltage-gated calcium channels maintain calcium homeostasis under fluctuating precipitation and temperature stress [38], representing subtle polygenic signals potentially overlooked by RDA. This difference supports previous evidence that LFMM is more sensitive for detecting small-effect ion-channel-related loci [39], whereas RDA is more powerful in capturing broad-scale genome-wide genetic structuring driven by composite environmental filtering [40]. Overall, the consistent enrichment of protein binding, ATP-dependent energy metabolism, and calcium ion transduction pathways in both RDA and LFMM highlights a conserved polygenic adaptation strategy maintaining cellular homeostasis, energy balance, and stress responses under arid and continental climates. The unique identification of voltage-gated calcium channel-related terms by LFMM further demonstrates the complementary value of combined GEA approaches in uncovering fine-scale ion-regulatory variation. Collectively, these convergent and distinct gene sets reveal a layered genomic architecture underlying multi-stress climate adaptation in local sheep breeds.

Our LFMM outputs highlight candidate genes that potentially underpin the coordinated adaptation of XH to alpine cold, drought and hypoxia, pointing to polygenic adaptive architectures analogous to those reported for other plateau ruminants [41], although functional validation remains required to confirm causality. Acting as the upstream molecular switch converting ambient climatic fluctuations into intracellular transcriptional responses, neural sensing genes *NGEF*, *DGKB*, *PIEZO2* and *EPHA6* may collectively contribute to organismal acclimatization. *NGEF*, which modulates neuronal sensitivity to prolonged cold and drought, carries climate-associated variants previously detected in climate-adapted indigenous sheep [6]; these variants could potentially enhance neuronal sensing of drought and temperature fluctuations through Rho-mediated osmotic adaptation and thermal signaling regulation [42]. *DGKB* governs lipid messenger turnover to transduce osmotic and thermal cues via an evolutionarily conserved ruminant stress pathway [43], and variants within this gene may modulate these signaling processes. The mechanosensitive channel *PIEZO2*, whose variants underpin cross-species altitude adaptation in sheep and goats, regulates respiration and vascular homeostasis against hypobaric stress [44], suggesting that sequence variation at this locus might facilitate highland acclimatization, while *EPHA6* drives adaptive neurovascular remodeling consistent with genomic selection footprints in highland sheep breeds [45]. Following environmental signal perception, metabolic genes *PPARGC1A*, *IGFBP7* and *GYS1* may reshape energy metabolism to sustain fitness under nutrient-limited alpine conditions. *PPARGC1A*, which serves as a master regulator of mitochondrial biogenesis and cold-triggered thermogenesis, exhibits temperature-linked polymorphisms in yaks and high-altitude cattle [46], implying its potential involvement in thermal adaptation. *IGFBP7* optimizes energy allocation under combined cold and drought stress [47], and *GYS1* promotes glycogen storage for cold tolerance in high-altitude sheep [48]; variants in these genes could support survival under alpine stress. To alleviate hypoxia-induced stress, *FARP1* and *DLC1* maintain cardiovascular integrity; FARP1 regulates cytoskeletal remodeling against hypoxic endothelial damage [49], whereas *DLC1* restrains aberrant RhoA signaling to prevent pathological vascular fibrosis [50], and associated variants may modulate these protective functions. *YARS1* and *TRAM2* preserve proteome stability under oxidative, osmotic, and hypoxic stress. *YARS1* maintains accurate translation under ultraviolet and drought stress, whereas *TRAM2* alleviates endoplasmic reticulum stress caused by extreme environments, consistent with Tibetan sheep multi-omic signatures [51]; sequence variants may fine-tune these cellular protective processes. For genome and cellular protection, *ALDH7A1* eliminates lipid-peroxidation-derived toxic aldehydes to maintain osmotic balance in arid highlands [52], and *RAD51B* repairs hypoxia- and UV-induced DNA double-strand breaks that threaten genomic integrity during population adaptive evolution [53]. Beyond stress defense, calcium channel genes and *ENPP2* propagate extracellular climatic signals via calcium second-messenger cascades, with *ENPP2* regulating endothelial and osmotic homeostasis consistent with yak cardiovascular adaptive transcriptomic evidence [46], indicating its potential role in climatic acclimatization. Meanwhile, immune modulator *SIAE* curbs excessive inflammation stimulated by alpine stressors to balance immune expenditure and survival fitness [41]. Taken together, LFMM-derived candidates complement RDA-based findings. Shared enrichment of protein-binding, ATP-binding and calcium-ion-binding pathways suggests that multi-system physiological coordination constitutes a core adaptive strategy for coping with drought and temperature stress; functional experiments are still needed to verify these hypothesized mechanisms.

Since global climate change is characterized by increasing fluctuations in temperature and precipitation patterns, it is expected to profoundly affect livestock production systems worldwide [54]. In this study, we integrated RONA and genetic offset analyses to estimate projected genomic mismatch as an indicator of potential maladaptive risk of indigenous Chinese sheep breeds under 12 future climate scenarios. We note that these metrics do not directly quantify adaptive capacity or climate resilience, and they cannot account for phenotypic plasticity, husbandry practices and other ecological factors. Our projections indicate that XH and CLH carry the highest maladaptation risk under future climate scenarios, consistent with theory that strongly locally adapted populations may suffer elevated genomic mismatch under rapid environmental change [55,56]. XH faces combined warming and precipitation reduction, with the SSP370 scenario (2081–2100) yielding the largest RONA and genetic-offset values. By contrast, BYBLK and SNT show relatively low projected genomic mismatch, likely owing to their broad distribution ranges and higher standing genetic diversity [57]. These differences offer insights to inform breed-specific conservation strategies. Breeds exhibiting high projected genomic mismatch such as XH and CLH should receive priority for germplasm preservation and targeted improvement through marker-assisted selection for drought and heat tolerance, whereas breeds with limited genomic mismatch such as BYBLK and SNT are more suitable for in situ conservation and sustainable utilization [58]. Overall, this heterogeneity in projected genomic mismatch among indigenous sheep breeds highlights the necessity of integrating genetic adaptive evidence into targeted germplasm conservation and breeding programs, which is essential for safeguarding the long-term stability and resilience of regional pastoral ecosystems under accelerating climate change.

However, this study still has several inherent limitations. Firstly, the future predictions in this research are established merely based on the statistical associations between SNP loci and climatic variables, without fully incorporating adaptive mechanisms related to genomic structural variations, epigenetic modifications, and gene expression regulation. Secondly, XH was the core breed targeted by our funded research on alpine-arid adaptation and we were able to collect a large cohort of unrelated individuals from specialized breeding farms, while only 9–10 pedigree-confirmed unrelated samples were obtained for the other five breeds due to limited accessible germplasm resources, meaning this pronounced imbalance may bring slight statistical noise even with corrective analytical approaches. Notably, despite the implemented strict quality-control procedures and bias-correction strategies for allele frequency calculation and SNP filtering, this unequal sampling size may still reduce the statistical power for the five breeds with smaller sample cohorts, for which we plan to expand sampling in follow-up studies to verify our findings. Thirdly, both RONA and genetic offset analyses in this study rely on the assumption that genotype–environment relationships are temporally stable, which may not hold true under rapid climate change and thus requires further rigorous verification [59]. Additionally, discrepancies among predictions from different climate models bring inherent uncertainties to our results, limiting the comprehensiveness and accuracy of the current adaptive assessment. Fourthly, we did not validate the identified climate-adaptive loci using independent external sheep populations, nor did we systematically compare our candidate variants with previously published ovine selection signatures. The absence of cross-population verification and inter-study signature comparison restricts the generalizability of our detected adaptive genes and genomic signals. To compensate for these deficiencies, future studies should expand sampling of small-cohort breeds, integrate transcriptomic and epigenomic data to unravel the multi-layered molecular mechanisms of climate adaptation, perform independent population validation for our adaptive loci, and conduct comparative analysis with published sheep selection signatures. Moreover, adopting multi-model ensemble prediction and cross-validation strategies can effectively reduce systematic biases of individual models, facilitating the construction of more robust and comprehensive predictive models to accurately evaluate the adaptive responses of sheep to future climatic stresses.

## 5. Conclusions

This study elucidated the genomic basis of climate-driven adaptive differentiation in indigenous sheep breeds via multi-omics landscape genomic analysis. Our model outputs suggest that temperature variability exhibits stronger statistical associations with ovine genetic divergence relative to water availability within the examined set of environmental variables; Continentality and minTempWarmest emerged as key environmental predictors, while precipitation seasonality (BIO14/BIO15) and aridity-related indices (aridityIndexThornthwaite, PETDriestQuarter) serve as vital secondary predictors. Combined GEA analyses identified conserved functional pathways including energy metabolism and calcium ion transduction, underlying the polygenic adaptation of sheep to arid and fluctuating continental climates. Future climate simulations revealed obvious spatial heterogeneity in projected genomic mismatch: western narrow-niche sheep breeds suffer severe genomic maladaptation under high-emission scenarios, whereas eastern populations possess stronger climatic resilience. Collectively, these findings deepen the systematic understanding of ovine climate adaptation mechanisms and offer a robust theoretical foundation and targeted guidance for the precision conservation of indigenous sheep germplasm resources and the directional cultivation of climate-resilient sheep varieties amid ongoing global climate change.

## Figures and Tables

**Table 1:** Description of environmental variables.

Variable Abbreviation	Full Name	Description
aridityIndexThornthwaite	Thornthwaite aridity index	Aridity index measuring the degree of water deficit
BIO14	Precipitation of driest month	Precipitation of the driest month
BIO15	Precipitation seasonality	Coefficient of variation in precipitation seasonality
Continentality	Continentality	Temperature difference between the warmest and coldest monthly mean values
minTempWarmest	Min temperature of warmest month	Minimum temperature of the warmest month
PETDriestQuarter	PET of driest quarter	Mean potential evapotranspiration of the driest quarter

**Table 2:** Significance test results and explained variance of RDA constrained axes.

Constrained Axis	Eigenvalue	Explained Variance Ratio	Cumulative Variance Ratio	df	Variance	F Value	*p* Value
RDA1	11,589.61	0.20	0.20	1	11,589.61	1.58	<0.001
RDA2	10,259.14	0.18	0.38	1	10,259.14	1.42	<0.001
RDA3	9847.87	0.17	0.56	1	9847.87	1.38	<0.001
RDA4	8699.89	0.15	0.71	1	8699.89	1.23	<0.001
RDA5	8390.36	0.14	0.86	1	8390.36	1.20	<0.001
RDA6	7465.06	0.13	1.00	1	7465.06	1.08	<0.001

**Table 3:** Significance test results and variance inflation factors of environmental variables in RDA.

Environmental Variable	VIF	df	Variance	F Value	*p* Value
aridityIndexThornthwaite	165.07	1	10,439.18	1.43	<0.001
BIO14	21.25	1	9347.06	1.28	<0.001
BIO15	36.08	1	8329.10	1.14	<0.001
continentality	36.19	1	9649.72	1.32	<0.001
minTempWarmest	88.72	1	8356.10	1.14	<0.001
PETDriestQuarter	4.21	1	10,130.80	1.39	<0.001

**Table 4:** Number of RDA candidate SNPs associated with each environmental variable.

Environmental Variable	Number of Candidate SNPs
aridityIndexThornthwaite	842
BIO14	1008
BIO15	668
Continentality	1653
minTempWarmest	314
PETDriestQuarter	3637

**Table 5:** Number of LFMM candidate SNPs associated with each environmental variable.

Environmental Variable	Number of Candidate SNPs
aridityIndexThornthwaite	1204
BIO14	1368
BIO15	912
Continentality	1107
minTempWarmest	708
PETDriestQuarter	2156

## Data Availability

The whole-genome resequencing raw data generated from our previous project have been deposited and are publicly accessible in the National Genomics Data Center, China National Center for Bioinformation (CNCB-NGDC, https://ngdc.cncb.ac.cn) under the project accession number PRJCA052911. Additional publicly available sheep whole-genome sequencing datasets were further retrieved from the National Center for Biotechnology Information (NCBI) database (www.ncbi.nlm.nih.gov, Table S2). All relevant data supporting the findings of this study are included within the manuscript and its Supplementary Files.

## Supplementary Materials

The following supporting information can be downloaded at: https://www.mdpi.com/article/10.3390/ani16172673/s1, Figure S1. High-quality photographs of each indigenous sheep breed and geographical map of sampling locations. (a) Photographs of the six studied sheep breeds. (b) Geographical map showing the sampling regions of all sheep populations included in this study; Figure S2. Population structure and LFMM association diagnostics. (a) ADMIXTURE ancestry proportions of individuals from six sheep breeds estimated at the optimal K = 4. (b) QQ plots of −log10-transformed *p*-values derived from LFMM analyses for the six environmental variables. λ denotes the genomic inflation factor calculated for each climate predictor; Figure S3: RONA projections under ACCESS-CM2 SSP370. RONA values for six climatic variables under ACCESS-CM2 SSP370 in (a) 2061–2080 and (b) 2081–2100; Figure S4: RONA projections under BCC-CSM2-MR scenarios. RONA values for six climatic variables under BCC-CSM2-MR: (a,b) SSP126; (c,d) SSP370. Time periods: 2061–2080 (a,c) and 2081–2100 (b,d); Figure S5: RONA projections under GISS-E2-1-G scenarios. RONA values for six climatic variables under GISS-E2-1-G: (a,b) SSP126; (c,d) SSP370. Time periods: 2061–2080 (a,c) and 2081–2100 (b,d); Figure S6. Mean RONA ± inter-model standard error (SE) for six populations under SSP126 and SSP370 climate scenarios during 2061–2080 and 2081–2100. Each panel corresponds to one environmental variable. Cyan dots = SSP126; red dots = SSP370. Error bars represent inter-GCM SE, reflecting uncertainty originating from different CMIP6 climate model simulations; Table S1: Pairwise kinship coefficients of sample pairs. Table S2: Summary of sheep whole-genome sequencing datasets retrieved from the NCBI database; Table S3: Sequencing statistics for each sample, summarizing raw read quality metrics and filtered clean read information.

## Abbreviations

The following abbreviations are used in this manuscript:

RDA	Redundancy analysis
LFMM	Latent factor mixed model
RONA	Risk of non-adaptedness
SNP	Single-nucleotide polymorphism
XH	Xiahe sheep
BYBLK	Bayinbuluke sheep
CLH	Cele Black sheep
SNT	Sunite sheep
HU	Hu sheep
STH	Small-tailed Han sheep
VIF	Variance inflation factor
GO	Gene Ontology
FDR	False discovery rate
GEA	Genotype–environment association
