# Supplementary material for: Landscape Genomics Reveals Divergent Adaptation Modes and Predicts Climate Vulnerability in Xinjiang Indigenous Sheep
Source: Animals (Basel). 2026 Aug 25;16(17):2673. doi: 10.3390/ani16172673 (PMC13564338; doi:10.3390/ani16172673)
Supplement: Supplementary file 1 [file animals-16-02673-s001.zip › Supplementary Tables/Table S1.pdf]

Table S1 Pairwise kinship coefficients of sample pairs.

Sample1	Sample2	relatedness_phi
STH06	STH10	0.0480016
STH10	STH06	0.0480016
STH06	STH08	0.0508985
STH08	STH06	0.0508985
STH06	STH09	0.0527842
STH09	STH06	0.0527842
STH07	STH10	0.101434
STH10	STH07	0.101434
STH07	STH09	0.103997
STH09	STH07	0.103997
STH07	STH08	0.104136
STH08	STH07	0.104136
STH09	STH10	0.160275
STH10	STH09	0.160275
STH08	STH10	0.160482
STH10	STH08	0.160482
STH08	STH09	0.163809
STH09	STH08	0.163809

Note: Kinship coefficients (relatedness_phi) were calculated by vcftools.
