# Supplementary material for: Landscape Genomics Reveals Divergent Adaptation Modes and Predicts Climate Vulnerability in Xinjiang Indigenous Sheep
Source: Animals (Basel). 2026 Aug 25;16(17):2673. doi: 10.3390/ani16172673 (PMC13564338; doi:10.3390/ani16172673)
Supplement: Supplementary file 1 [file animals-16-02673-s001.zip › Supplementary Tables/Table S2.pdf]

Table S2 Summary of sheep whole-genome sequencing datasets retrieved from the NCBI database.

Sample	Run	BioProject	sex	Breed
HU01	SRR16896207	PRJNA779188	male	Hu
HU02	SRR16896149	PRJNA779188	male	Hu
HU03	SRR16896166	PRJNA779188	male	Hu
HU04	SRR16896192	PRJNA779188	male	Hu
HU05	SRR16896191	PRJNA779188	male	Hu
HU06	SRR16896218	PRJNA779188	male	Hu
HU07	SRR16896224	PRJNA779188	male	Hu
HU08	SRR16896204	PRJNA779188	male	Hu
HU09	SRR16896167	PRJNA779188	male	Hu
HU10	SRR16896203	PRJNA779188	male	Hu
STH02	SRR8560981	PRJNA521847	female	Small-tailed Han
STH03	SRR8560982	PRJNA521847	female	Small-tailed Han
STH04	SRR8560979	PRJNA521847	female	Small-tailed Han
STH05	SRR8560980	PRJNA521847	female	Small-tailed Han
STH06	SRR8560984	PRJNA521847	female	Small-tailed Han
STH07	SRR5149614	PRJNA357880	female	Small-tailed Han
STH08	SRR11657682	PRJNA624020	female	Small-tailed Han
STH09	SRR11657497	PRJNA624020	female	Small-tailed Han
STH10	SRR11657584	PRJNA624020	female	Small-tailed Han
SNT1	SRR8560961	PRJNA521847	female	Sunite
SNT2	SRR8560973	PRJNA521847	female	Sunite
SNT3	SRR8560965	PRJNA521847	female	Sunite
SNT4	SRR8560967	PRJNA521847	female	Sunite
SNT5	SRR8560964	PRJNA521847	female	Sunite
